# Apolipoprotein A-II Plus Lipid Emulsion Enhance Cell Growth via SR-B1 and Target Pancreatic Cancer *In Vitro* and *In Vivo*

**DOI:** 10.1371/journal.pone.0151475

**Published:** 2016-03-22

**Authors:** Sohel M. Julovi, Aiqun Xue, Thao N. Thanh LE, Anthony J. Gill, Jerikho C. Bulanadi, Mili Patel, Lynne J. Waddington, Kerry-Anne Rye, Minoo J. Moghaddam, Ross C. Smith

**Affiliations:** 1 Cancer Surgery Group, Kolling Institute of Medical Research, University of Sydney at Royal North Shore Hospital, St Leonards, New South Wales, Australia; 2 Department of Gastrointestinal Surgery, Royal North Shore Hospital, St Leonards, New South Wales, Australia; 3 Cancer Diagnosis and Pathology Research Group, Kolling Institute of Medical Research,University of Sydney at Royal North Shore Hospital, St Leonards, New South Wales, Australia; 4 CSIRO Manufacturing, CSIRO, North Ryde, New South Wales, Australia; 5 CSIRO Manufacturing, CSIRO, Parkville, Victoria, Australia; 6 Centre for Vascular Research, Faculty of Medicine, University of New South Wales, New South Wales, Australia; Beijing Key Laboratory of Diabetes Prevention and Research, CHINA

## Abstract

**Background:**

Apolipoprotein A-II (ApoA-II) is down regulated in the sera of pancreatic ductal adenocarcinoma (PDAC) patients, which may be due to increase utilization of high density lipoprotein (HDL) lipid by pancreatic cancer tissue. This study examined the influence of exogenous ApoA-II on lipid uptake and cell growth in pancreatic cancer (PC) both *in vitro* and *in vivo*.

**Methods:**

Cryo transmission electron microscopy (TEM) examined ApoA-II’s influence on morphology of SMOFLipid emulsion. The influence of ApoA-II on proliferation of cancer cell lines was determined by incubating them with lipid+/-ApoA-II and anti-SR-B1 antibody. Lipid was labeled with the fluorophore, DiD, to trace lipid uptake by cancer cells *in vitro* by confocal microscopy and *in vivo* in PDAC patient derived xenograft tumours (PDXT) by fluorescence imaging. Scavenger receptor class B type-1(SR-B1) expression in PDAC cell lines and in PDAC PDXT was measured by western blotting and immunohistochemistry, respectively.

**Results:**

ApoA-II spontaneously converted lipid emulsion into very small unilamellar rHDL like vesicles (rHDL/A-II) and enhanced lipid uptake in PANC-1, CFPAC-1 and primary tumour cells as shown by confocal microscopy. SR-B1 expression was 13.2, 10.6, 3.1 and 2.3 fold higher in PANC-1, MIAPaCa-2, CFPAC-1 and BxPC3 cell lines than the normal pancreatic cell line (HPDE6) and 3.7 fold greater in PDAC tissue than in normal pancreas. ApoA-II plus lipid significantly increased the uptake of labeled lipid and promoted cell growth in PANC-1, MIAPaCa-2, CFPAC-1 and BxPC3 cells which was inhibited by anti SR-B1 antibody. Further, ApoA-II increased the uptake of lipid in xenografts by 3.4 fold.

**Conclusion:**

Our data suggest that ApoA-II enhance targeting potential of lipid in pancreatic cancer which may have imaging and drug delivery potentialities.

## Introduction

Serum apolipoprotein A-II (ApoA-II) was found to be depressed in pancreatic cancer patients and was a potential diagnostic biomarker [1]. This work has been confirmed by Honda and colleagues [2] and others [3]. ApoA-II is a component of high density lipoprotein (HDL) where it has an important role in directing the fate of the metabolism of the lipid in the HDL. The most important role for HDL is the delivery of cholesterol from peripheral tissues to the liver where it is metabolised into bile salts or excreted to the bile. The scavenger receptor class B type-1 (SR-B1) is prevalent in hepatocytes and attracts HDL where the lipid is endocytosed [4, 5] or diffused [6, 7] in hepatocytes.

SR-B1 is a multi-ligand receptor and highly expressed in a variety of tumor cells including prostate, breast, colorectal and ovarian cancer cells [8]. The lipoprotein on the surface of HDL binds to SR-B1and delivers its lipid into the cell through a pore formed by SR-B1 increasing the uptake of HDL-cholesteryl ester (CE) [9], an essential nutrient for malignant cell proliferation and metastasis [10, 11]. Thirty percent of HDL is associated with ApoA-II which is thought to make the HDL smaller than that with ApoA-I alone and makes the HDL/ApoA-II less attracted to the hepatic SR-B1 [12]. In addition, ApoA-II maintains HDL levels in part by inhibition of hepatic lipase [13]. These results in a relatively longer circulation time for HDL/ApoA-II. ApoA-II alters binding of HDL to SR-B1 in different tissues and is particularly attracted to steroidogenic tissue [14] where the cholesterol is utilized for hormone synthesis but rapidly growing cancer cells also require cholesterol for cell membranes [15]. Cancer cells have an increased expression of SR-B1in relation to the expression on their non-malignant cells of origin [16, 17] and it is possible that this results in increased uptake of HDL by the cancer tissue which is an explanation for the reduction in serum ApoA-II in PDAC cases [1–3]. Interestingly pancreatic cancer does not accumulate fludeoxyglucose (FDG) strongly enough to be reliable as a contrast agent for Positron Emission Tomography (PET) [18] which probably indicates that pancreatic cancer has a preference for lipid as its main calorie source [19]. If lipid is the main source of energy for pancreatic cancer tissue it is possible that HDL is an important source of this lipid. Energy metabolism of the cells is involved in complex carcinogenesis [20, 21]. The role of cholesterol metabolism in the process of carcinogenesis is also reported [22–24].Cancer and other rapidly proliferating cells require cholesterol and other membrane components to optimize growth [25]. HDLs have been implicated in cholesterol delivery in some malignancies, including breast cancer [26], ovarian cancer [8], adrenocortical tumours [27] and prostate cancer [28]. It is likely that pancreatic cancer is also avidly utilsing HDL.

The hypothesis tested in this paper is that ApoA-II will expedite the uptake of lipid into pancreatic cancer tissue and will thus increase cell growth. Further, we hypothesize that the level of expression of SR-B1 will correlate with the uptake of lipid by pancreatic cancer cells and will be greater than that of normal tissue.

## Materials and Methods

### Cell culture

PANC-1, MIAPaCa-2, BxPC3 and CFPAC-1 pancreatic cancer cell lines were gifts from Prof. Barry Allen (St George Hospital, NSW, Australia), and normal human pancreatic ductal epithelial (HPDE6) cells [29] were from Dr. Chris Scarlett (School of Environmental & Life Sciences, University of Newcastle, NSW, Australia). A549 lung cancer cell line, T47D and MCF7 breast cancer cell lines were kindly provided by Prof. Ross Davey (Kolling Institute of Medical Research, Royal North Shore Hospital, NSW, Australia). PC3 prostate cancer cell line was kindly provided by Prof. Qihan Dong (Central Clinical School, Bosch Institute, The University of Sydney, NSW, Australia). Except HPDE6 cell line, all cell lines were purchased from ATCC. Cell lines were typed by short tandem repeat profiling and they conformed to the ATCC reference standards (CellBank, Westmead, NSW, Australia). BxPC3, CFPAC-1, A549, T47D and MCF7 cell lines were cultured in RPMI 1640, PANC-1and MIAPaCa-2 were cultured in Dulbecco’s Modified Eagle Medium (DMEM) and PC3 prostate cells were cultured in F-12K (Gibco, BRL) with 10% FBS, containing 10% fetal bovine serum (FBS; ICN, Aurora, OH) in 75 cm^2^ flasks at 37°C in a humidified 5% CO_2_ atmosphere. HPDE6 cells were cultured in keratinocyte serum-free (KSF) medium supplemented by epidermal growth factor and bovine pituitary extract (Life Technologies, Inc., Grand Island, NY).

### Primary culture

Human pancreatic tumour specimens were obtained at the Royal North Shore Hospital (Sydney, Australia), with their informed written consent, following protocols approved by the Northern Sydney Local Health District Human Research Ethics Committee (NSLHD HREC) (Protocol number: 0909-227M, Sydney, Australia). Primary tumour cell cultures were established by explant growth of small fragments (≈1 mm) dissected from the PDAC tumour tissues, placed in DMEM (pH7.4) supplemented with 10% heat-inactivated FBS with 50 units/ml penicillin/streptomycin. Cultures were used after passage 3 to avoid residual contamination by macrophages [30]. All cultures were collected under identical growth and serum conditions. We used 24 h serum starved cells for experiments to avoid the early gene expression induced by serum.

### Labeling of SMOF lipid with the fluorophore DiD

The SMOF lipid emulsion that contains soya oil, medium chain triglyceride, olive oil and fish oil {www.tga.gov.au/pdf/auspar/auspar-smoflipid.pdf}, and is used for intravenous nutrition, was used in our experiments after labeling with a 0.05% lipophilic tracer fluorophore, DiD, (DiD307, Invitrogen, Carlsbad, CA). The SMOF lipid emulsion was dried under a rotary evaporator, freeze-dried before mixing well with DiD lipid in an ethanolic solution. The ethanol was finally evaporated and the residual lipid film was rehydrated by addition of sterile Baxter water to make the final concentration to 20% SMOFlipid, followed by harsh vortexing and 30 minutes ultrasonic bath homogenization.

### Collection and purification of ApoA-II

ApoA-II was purified as described in previous studies [31–33]. HDLs were ultracentrifugally isolated from pooled samples of normal human plasma (1.063<d<1.21 g/ml)and delipidated. The resulting apoHDL was subjected to chromatography on a Q-Speharose Fast Flow column attached to an Akta FPLC system (GE Healthcare, Chalfont St Giles, Buck, UK). Fractions containing apoA-II were pooled and purity was checked by SDS-PAGE and Coommassie staining, which revealed a single band. The pooled samples were lyophilised, reconstituted with 3 M guanidine hydrochloride/10 mM Tris/0.01% (wt/v) EDTA-Na_2_, and dialysed into endotoxin-free PBS prior to use.

### Cell proliferation assay

Cell proliferation was performed in different cell lines and quantified by the crystal violet assay as described in previously [34], where MTT assay is not suitable in lipid treated cells [35]. 4×10^4^ cells/ml cells were seeded in ninety six well microplates, for all cell lines except for PANC-1 and MIAPaCa-2 where 2x10^4^ cells/ml were seeded to a final volume of 200μl. Serum free cells were treated with or without SMOFlipid (lipid) alone, ApoA-II alone and or in combination in different concentrations and incubated for 24 h, 48 h and 72 h. To test the effect of SR-B1, cells were pretreated with anti SR-B1 (2.5μg/ml) for 2 h and washed by media 3 times for 5 minutes each. Then the cells were treated with or without lipid plus ApoA-II for 48 h. Culture medium were removed and stained with 1 μg/ml crystal violet (Sigma Aldrich, St. Louis, MO) dissolved in 25% methanol (v/v) and 75% distilled water (v/v). Bound crystal violet was solubilised with 0.1% sodium-dodecyl-sulphate (SDS) in phosphate buffered saline (PBS). The optical density of each well was determined at the 550 nm wavelength. Results were expressed as relative to control. We found that 1:30 dilution of lipid and 50 μg/ml of ApoA-II were the optimum concentrations at 48 h and this condition was used in the subsequent study.

### Immunoblotting

Immunoblot analysis was performed as described previously [1]. Briefly, 4 x10^5^ cells of each cell lines seeded in 6 well plates and cells were grown until confluent. Equal amounts of proteins were separated by 10% SDS-PAGE under reducing conditions and transferred to a polyvinylidene difluoride membrane. The primary antibody used was Rabbit anti- SR-B1 (Novus Biologicals, Littleton, USA). Immunoreactivity was detected with the electrochemiluminescence detection system (Amersham Biosciences, Buckinghamshire, UK). Anti-human β-actin antibody was included to normalize against unequal loading.

### Confocal microscopy

The uptake of SMOFlipid labeled with DiD was investigated by Confocal microscopy. Briefly, 1x10^5^ cells/well were seeded on 8 well culture slides (BD Falcon, BD bioscience) for overnight and treated with lipid labeled with DiD dye at 1:30 dilution with or without ApoA-II for 48 h. In some experiments cells were pre-incubated with the1:40, 1:30 and 1:10 dilutions of unlabeled SMOFlipid and anti SR-B1 at (2.5 μg/ml) for 2 h, washed with serum free media and incubated with the lipid plus ApoA-II for 48h. Cells were fixed with 4% paraformaldehyde (Univar, Redmond,WA, USA), permeabilised with 0.3% Tween 20 (Sigma Aldrich, Castle Hills, NSW, Australia) and incubated with or without primary antibody, goat anti-human ApoA-II (Abcam, Abcam Inc., MA, USA) for 2 h and then incubated with green flurophore conjugated isotype matched secondary antibody (AlexaFlour^a^488 donkey anti goat IgG (H+L) (Invitrogen, Carlsbad, CA) for another 1 h at room temperature. Cells were mounted with glycerol media with DAPI (Invitrogen, Carlsbad, CA) and cellular uptake was examined under the Leica Confocal Microscopy (Leica, Tokyo, Japan).

For live cell imaging, 1x10^5^ cells/well were seeded on μ- Slide 4 well ibi Treat Microscopy Chamber (ibidi GmBh, Martinsried, Germany) for overnight and treated with lipid labeled with DiD dye at 1:20 and 1:10 dilution with or without ApoA-II for 24 h. Cellular uptake was examined under the Leica Confocal Microscopy (Leica, Tokyo, Japan). Lipid uptake intensity was quantified by the Leica Microsystem LAS AF (GmbH, Mannheim, Germany) and results were expressed as relative intensity.

### Cryo transmission electron microscopy (TEM)

A laboratory-built humidity-controlled vitrification system was used to prepare the samples for Cryo-TEM. Humidity was kept close to 80% for all experiments and ambient temperature was ∼22°C. A 4 μl aliquot of the sample was pipetted onto a 300 mesh copper grid coated with lacy formvar-carbon film (Pro-SciTech, Thuringowa, Queensland). After 30 s adsorption time, the grid was blotted manually using Whatman 541 filter paper, for between 2 and 10 s. The grid was then plunged into liquid ethane cooled by liquid nitrogen. Frozen grids were stored in liquid nitrogen until examined. The samples were examined using a Gatan 626 cryoholder (Gatan, Pleasanton, CA, USA) and a Tecnai 12 Transmission Electron Microscope (FEI, Eindhoven, The Netherlands) at an operating voltage of 120 kV, equipped with a FEI Eagle 4k × 4k CCD (FEI, Eindhoven, the Netherlands). Samples were viewed at 100 000–150 000 times magnification.

### Gel electrophoresis

An aqueous 0.5% m/m agarose gel was prepared and immersed in 1 × TBE (Tris-Borate-EDTA buffer, prepared by diluting 10x stock solutions, pH 9) buffer [36, 37], run in a horizontal electrophoresis system (Mini-Sub Cell GT, Biorad, electrode spacing 15 cm) for 45 min at 90 V. Gel images were taken with a Carestream MI at wavelength of 650 nm excitation and 700 nm emission.

ApoA-II plus lipid was reconstituted by the incubation of 2 μl ApoA-II (2mg/ml) and 2 μl of labeled SMOFlipid (10 mg/ml) at 37°C for 24 hr. Before loading the gel with the sample, each sample was diluted at 1:5 in PBS (pH 7.4) and added 2 μl of 6X loading dye solution (#R0611, Fermentas, Life Science) contain 10mM Tris-HCl (pH 7.6), 0.03% bromophenol blue, 0.03% xylene cyanol FF, 60% glycerol 60mM EDTA buffer. 12 μl of sample was loaded into each well of the gel. All of the experiments were performed at room temperature.

### Generation of PDAC patient derived tumour xenograft (PDTX) in non—obese diabetic/severe combined immunodeficient (NOD/SCID) mice

All animal experiments conformed to the guidelines of the Northern Sydney Local Health District (NSLHD) Animal Ethics Committee in this study and housed at Kearns facility, Kolling Institute of Medical Research, the University of Sydney. The protocol was approved by the NSLHD Animal ethics committee (Protocol number 1011-015A). All surgery was performed under anesthesia by using isoflurane with NO_2_ and Oxygen (2:1) and all efforts were made to minimize suffering. We have established PDTX model as described by Wong MH and Xue A et al. [38, 39] under the protocol NSLHD HREC- 0909-227M. Briefly, male NOD/SCID mice, ages 6 to 8 weeks, were anaesthetized using isoflourane with NO_2_ and oxygen (2:1). Small pieces of patient’s fresh tumour tissues were xenografted under the subrenal capsule (SRC) in a mouse. At eight weeks post-xenograft, mice were used to investigate the *in vivo* lipid uptake.

The animals bearing the first generation xenografts (F1) were randomized and distributed into three groups (a) vehicle (saline); (b) lipid (c) lipid plus ApoA-II and injected via tail vein. To homogenize the tumour volumes some of the F1 tumours were passage into other group of mice (F2) and subsequently passage into F3. The animal bearing F3 generation of xenograft tumours were used for validation study. Lipid uptake was measured at different time points by Carestream Molecular Imaging (MI) (Carestream Molecular Imaging, USA). At the end of each experiment organs and tumours were harvested and analysed further.

### In vivo image analysis

For in vivo optical imaging, tumor-bearing mice were injected with DiD307 labeled lipid with or without Apo-AII by tail vein injection. Imaging was performed immediately after injection, at 4 h, 24 h, 48 h and 96 h by in-vivo Carestream MI Imaging System. Mice were sacrificed at 48 h after injection, kidney’s with tumour, liver, spleen, pancreas, lungs, brain and muscle were harvested and analysed by Carestream MI at wavelength of 650 nm excitation and 700 nm emission.

### Histology and immunohistochemistry

Tissue samples were fixed in 10% phosphate buffered formalin and embedded in paraffin. Formalin-fixed, paraffin embedded sections were cut 4-μm thick sections and stained with Mayer’s hematoxylin and eosin (H & E). For immunohistochemistry, sections were incubated with anti-ApoA-II (# ab 54796, Abcam, Abcam Inc., MA, USA) (1 in 4000), anti-SR-B1 (#NB 400–104, Novus Biologicals, Littleton, USA) (1 in 4000) and isotype-matched IgG antibodies. Immunodetection was done using the Dako Envision + System-HRP labeled polymer Detection kit (Dako, Carpinteria, CA, USA) according to manufacturers' protocol and counterstained with Mayer's hematoxylin and Scott's bluing solution. After mounting, sections were observed under a light microscope (ECLIPSE 80i; Nikon, Tokyo, Japan).

### Statistical analysis

Data were expressed as mean ± standard deviation (S.D.). The statistical significance of group differences were analyzed by paired *t*-test and analysis of variance (ANOVA) followed by the Bonferroni post hoc test (where appropriate). Statistical significance was accepted at the *p* < 0.05 level.

## Results

### ApoA-II’s effect on SMOF lipid emulsion

**Fig 1:**
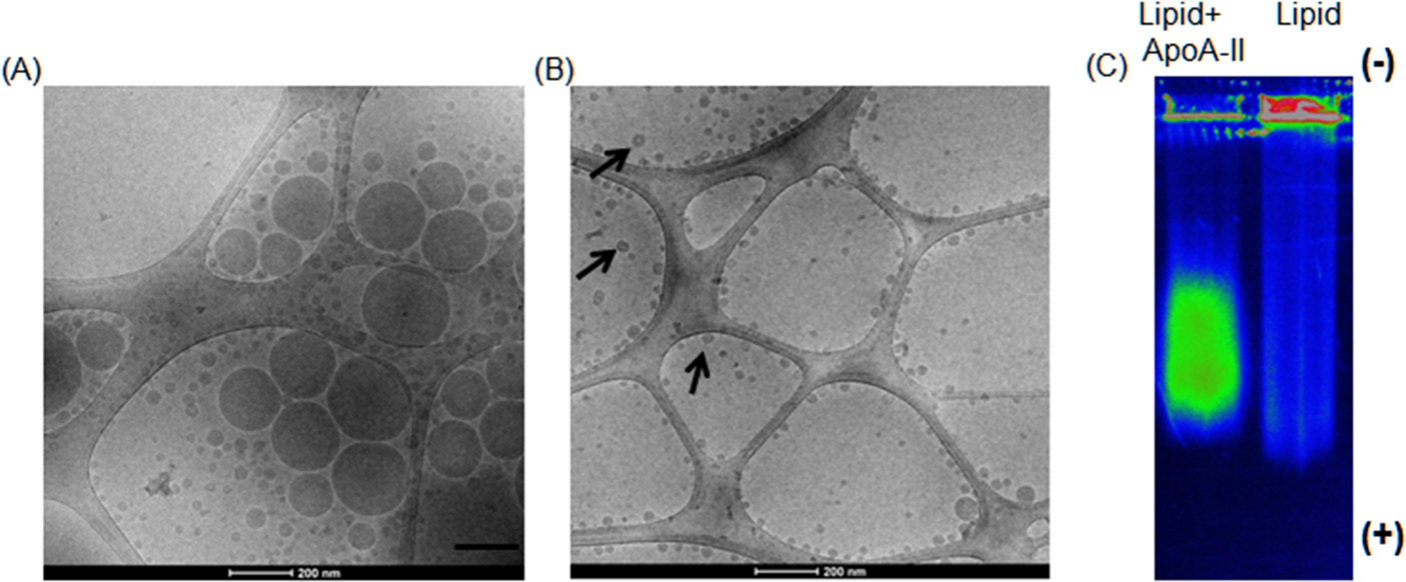
ApoA-II reduces the size of lipid. Cryo-TEM micrograph picture of the SMOFlipid emulsion without ApoA-II (A) and after addition of ApoA-II (B). Note the bi-layer structure of the lipid surface of the nanoparticle like structures in presence of ApoA-II (black arrows). (C) Spectral flourescence color photograph of a 0.5% agarose gel run for 45 min at 90 V in 1 × TBE buffer. SMOFlipid labeled with DiD did not migrate through the well but reconstituted labeled SMOFlipid with ApoA-II migrated as a band.

When ApoA-II was added to the opaque lipid emulsion, a phase transition occurred and the emulsified opaque solution transformed to a transparent clear liquid in less than one hour (S1 Fig). Cryo TEM images of SMOF lipid plus ApoA-II demonstrated that the emulsified particles transformed to unilaminar vesicles (Fig 1B). More interesting is the small size of these structures, many of which were 10–50 nm in diameter, far smaller than the original particles in the emulsion. This morphological change in structure and size are most likely due to the interaction and integration of the ApoA-AII within the lipid self-assemblies and the transformation to a transparent solution containing mostly small unilamellar vesicles, as demonstrated by Cryo-TEM (Fig 1A and 1B).

Gel electrophoresis demonstrated that reconstituted ApoA-II plus lipid complexes migrated through the gel as a band while lipid alone still stayed in the well (Fig 1C), suggesting that ApoA-II altered the lipid emulsion and reduced the particle size allowing migration into the gel. This is consistent with the cryo TEM results (Fig 1A and 1B).

### Confocal microscopy of cancer cells grown in lipid solutions with and without ApoA-II

**Fig 2:**
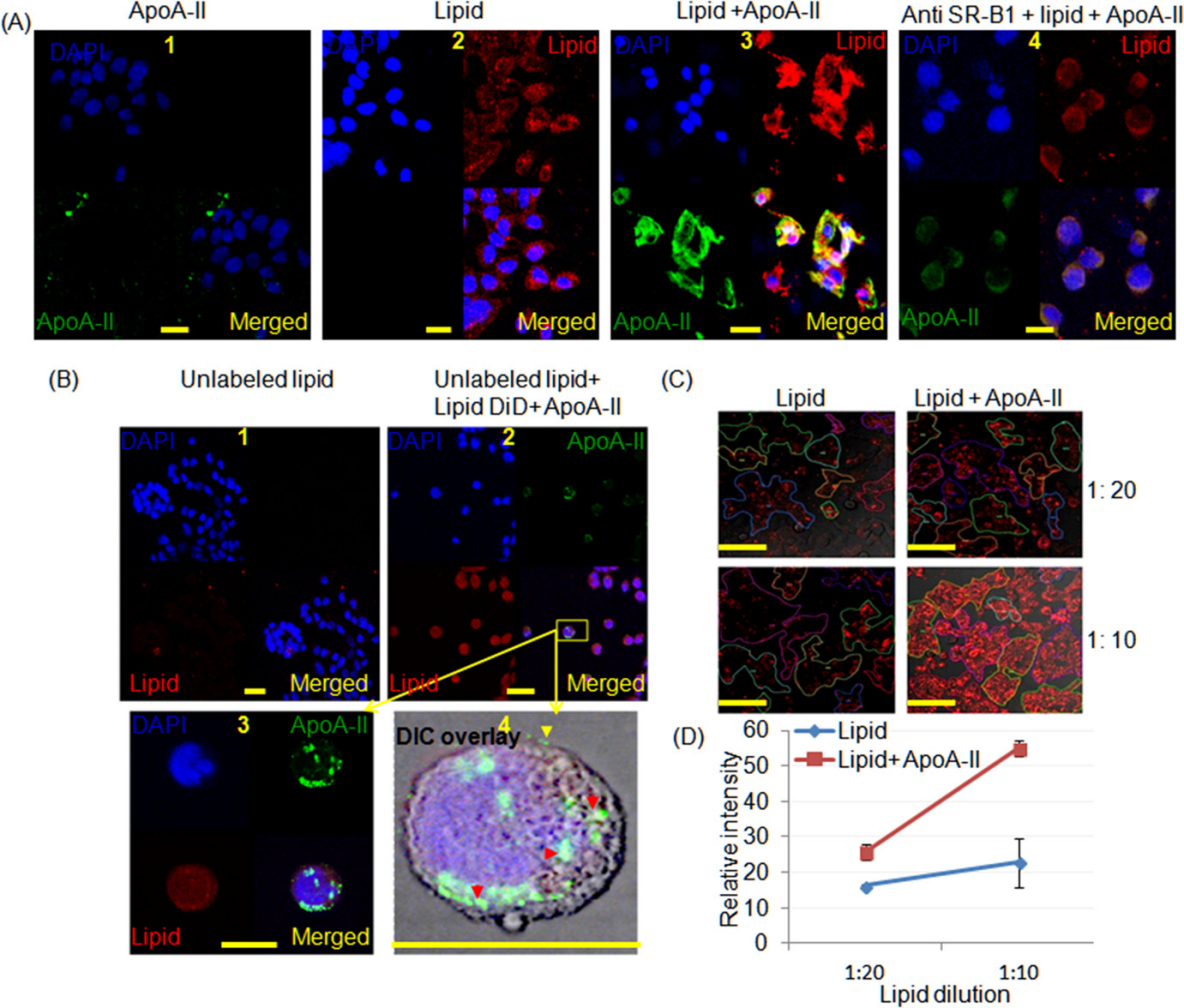
Confocal micrographs of CFPAC-1 cells demonstrating the influence of ApoA-II on lipid uptake. SMOFlipid was labelled with DiD (red), ApoA-II was labelled green with a secondary antibody and DAPI stained the nuclei blue. (A1) cells treated with ApoA-II alone, (A2) cells treated with lipid alone, (A3) cells treated with reconstituted lipoproteins after adding ApoA-II to DiD labeled SMOFlipid (SMOF/A-II) demonstrating increased uptake and (A4) cells treated with (SMOF/A-II) after pretreating with anti-SR-B1antibodies which reduced the lipid uptake (scale bar, 20 μm). (B) Cells were pretreated with unlabeled lipid at 1:10 dilution and (B2) after 2 h SMOF/A-II was added, it appeared to be endocytosed in cytoplasmic vesicles as shown in (B3) and (B4). ApoA-II (green) is attached with the cell membrane (yellow arrow head) and as well in the cytoplasm in part associated with the lipid (red arrow head) in Differential Interference Contrast (DIC) overlay image (scale bar 25 μm). (C) Live cell imaging experiment demonstrated greater uptake of lipid in cells when reconstituted lipoproteins after adding ApoA-II to DiD labeled SMOFlipid was added to the media for lipid concentrations of 1:20 and 1:10 (scale bar 100 μm). (D) The intensity of the DiD staining was greater when 4 samples were examined with eight to twelve areas of interest for each study where ApoA2 increased the uptake of lipid 25.6±2.2, *P* = 0.02 and 54.8±2.2, *P* = 0.004 for dilutions of 1:20 and 1:10 respectively.

The uptake of lipid by CFPAC-1 cells (Fig 2A3), PANC-1 (S2A Fig) and primary tumour cell lines (S2B Fig) was enhanced when reconstituted lipid plus ApoA-II was added to the culture medium. Similar effects were also seen in A549 lung cancer cells (S2C Fig). In these experiments ApoA-II was visualized by ApoA-II antibodies which demonstrated that it was localised to cancer cell membranes (Fig 2A and S2 Fig). While reduced fluorescence intensity was observed in CFPAC-1 cells when cells were pre incubated with the anti SR-B1 blocking antibody (Fig 2A4). When CFPAC-1 cells were pre incubated with the unlabeled large excess SMOFlipid alone (Fig 2B1) and then incubated with the reconstituted SMOFlipid/ApoA-II/DiD (Fig 2B2) further uptake of DiD occurred and the ApoA-II antibody stained peripheral components of cells. Thus although preincubation reduced the uptake of SMOFlipid/ApoA-II uptake still occurred through the mechanism of surface attraction to ApoA-II [4, 5]. In the presence of ApoA-II lipid uptake is significantly greater at various concentrations compared with the lipid alone (Fig 2C and 2D). These results suggest that ApoA-II is attracted to the cell membrane while lipid was transport to the PC cells. This finding is in an agreement with the Fan Y *et al*. [4] and Silver *et al*. [5]. Taken together these results support the proposition that lipid nanoparticle may be endocytosed.

### SMOFlipid ApoA-II targets PDAC PDTX in vivo

**Fig 3:**
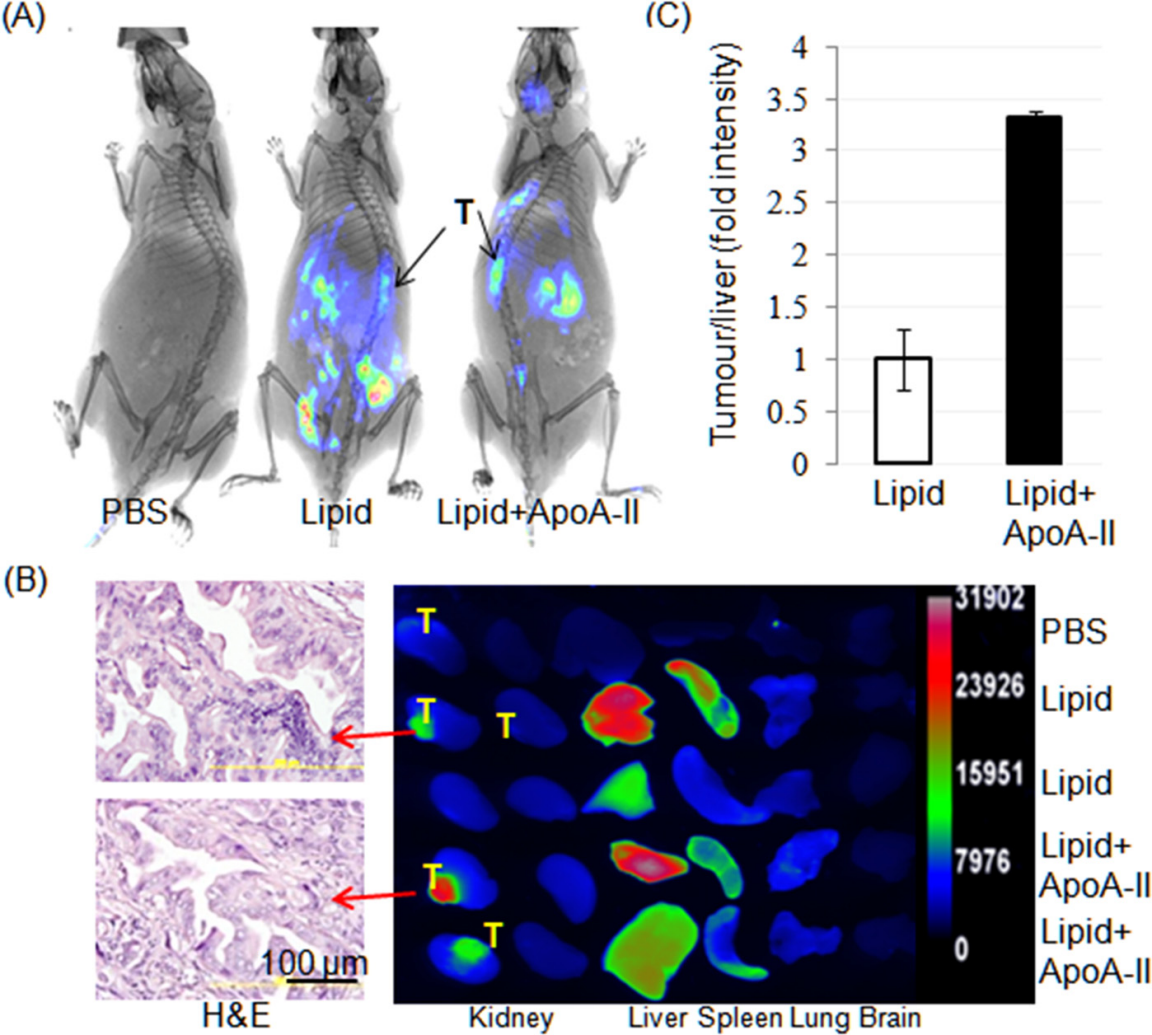
SMOFlipid ApoA-II targets pancreatic ductal adenocarcinoma (PDAC). (A) Spectral reflectance fluorescence images of 8 week old xenografts taken 48h after tail vein injection of PBS, DiD labeled SMOFlipid without or with ApoA-II using Carestream molecular imaging. Note the widespread distribution of DiD is better localized to the tumours when ApoA-II is included with lipid. Arrows indicate the site of tumours. (B) Spectral fluorescence images of explanted tumours and organs taken 48h after injection from mice after a tail vein injection of either PBS, lipid/DiD (n = 2) or lipid/DiD plus ApoA-II (n = 2) (right panel) along with the H&E photo micrograph of the tumour from two mice (left panel), retained the morphological characteristics with the original patient’s PC tumours in lipid and lipid plus ApoA-II treated mice. Note the uptake of fluorescence by the tumours, liver and spleen. (C) Graph represents the uptake by the tumour relative to the uptake of the liver as a fraction of area. These results were then normalised to the value for mice receiving lipid only (defined as 1) when the tumour uptake for the mice receiving lipid and ApoA-II had 3.4 fold increased uptake. Values are mean ± SD; *n* = 3.

To assess the tumor targeting capacity of lipid plus ApoA-II we employed a PDAC PDTX model in mouse. Lipid/DiD or lipid/DiD/ApoA-II was injected into the tail vein of first generation tumour bearing mice. Whole animal imaging after 48 h revealed lipid uptake in the PDAC xenografts tumours is confined in the lipid plus ApoA-II injected mice compared to lipid alone (Fig 3A and S3A Fig) but also in the liver, stomach and spleen. This was confirmed on the explanted organs by *ex vivo* imaging but little lipid was seen in lungs and brain but not seen in normal pancreas (Fig 3B and S3B Fig). The relative fluorescence intensity was increased by 3.4 fold in targeted cancer tissues, when DiD-lipid/ApoA-II was injected (Fig 3C) compared to DiD-lipid, consistent with Fig 2. Taken together our results suggest that ApoA-II targets human PDAC.

### Scavenger receptor B class type 1 (SR-B1) expression is higher human PDAC

**Fig 4:**
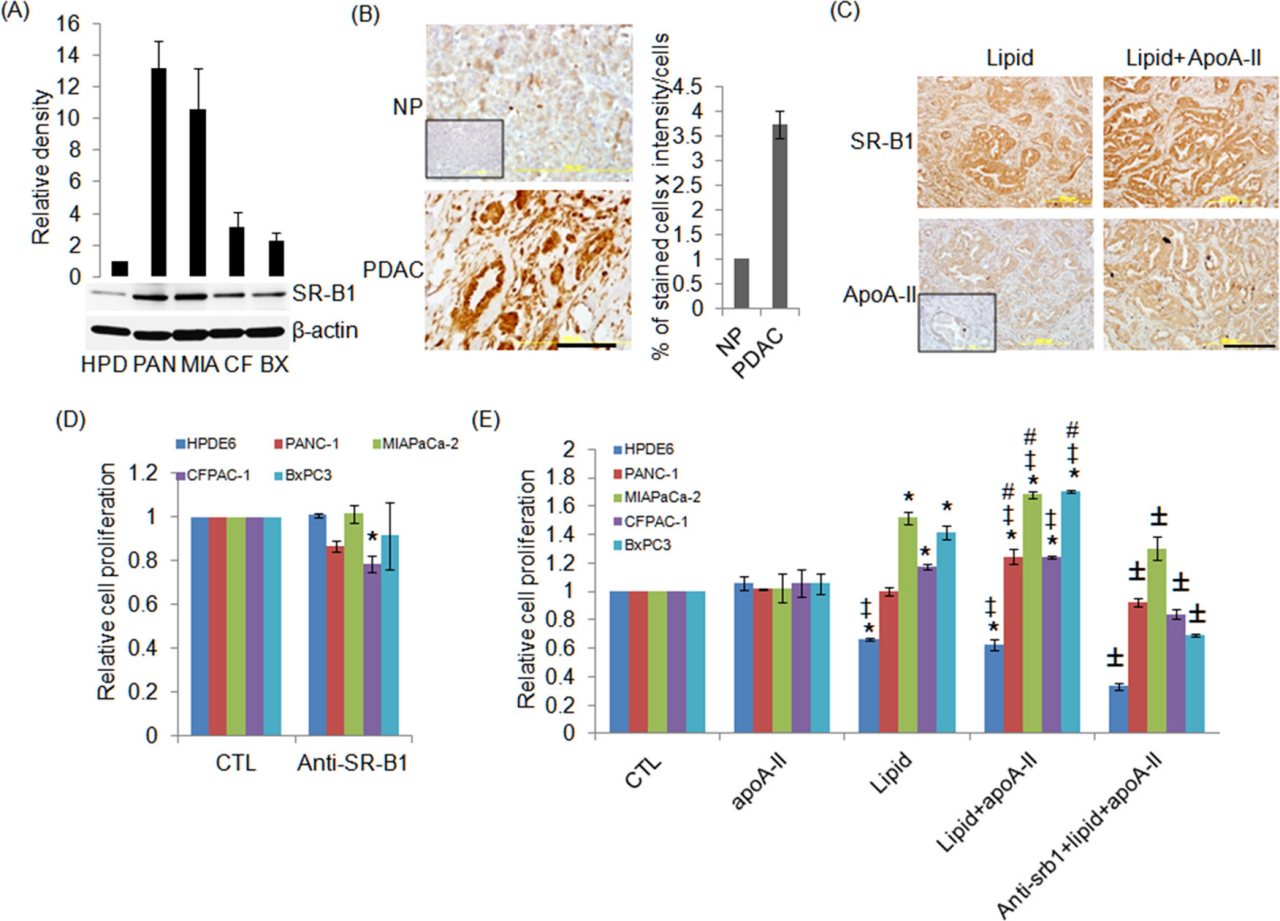
ApoA-II plus lipid increased lipid uptake and cell growth may be via SR-B1 in pancreatic cancer. (A) HPDE6, PANC-1, MIAPaCa-2, CFPAC-1 and BxPC3 cells were cultured and SR-B1 expression was measured by western blotting. The band intensity of the proteins was normalized with β-actin and HPDE6 was defined as 1. Values are the mean of two separate experiments. (B) Expression of SR-B1 by IHC on normal pancreas (NP) and human PDAC tissues. Inset, IgG represents the isotyped matched primary antibody to anti SR-B1. Graph shows the quantification of SR-B1 expression by the percentage of stained cells X intensity from 3 separate patients and NP was defined as 1. (C) Expression of SR-B1 and ApoA-II by IHC in corresponding xenografted PDAC tumours at 48 h after tail vein injection of lipid with or without ApoA-II. Inset, IgG represents the isotyped matched primary antibody to anti-ApoA-II. (D) Effects of anti SR-B1 alone in HPDE6, PANC-1, MIAPaCa-2, CFPAC-1 and BxPC3 cell growth. (E) Cells were pretreated with or without the SR-B1 antibody (2.5 μg/mL) for 2 hours and then treated with ApoA-II, lipid and lipid plus ApoA-II for 48 hours. Proliferation was measured by crystal violet assay. Values are mean ± SD; *n* = 4. *^, ‡, #^ and ± p< 0.05 vs. control, ApoA-II, lipid and lipid+ApoA-II respectively, with the use of analysis of variance.

By western blotting, for the first time, we demonstrated that SR-B1 expression was 13.2, 10.6, 3.1 and 2.3 fold higher in PANC-1, MIAPaCa-2, CFPAC-1 and BxPC3 cell lines respectively compared to normal pancreas cell line (HPDE6) (Fig 4A). Immunohistochemistry (IHC) demonstrated that SR-B1 expression was 3.7 fold greater in PDAC tissue compared to normal pancreas (Fig 4B). Consistent with Fig 2, we found that ApoA-II expression was higher in tumours when mice were injected with combination of lipid and ApoA-II emulsion (Fig 4C), while there was no difference in SR-B1 expression in tumours in any groups (Fig 4C).

### ApoA-II enhanced cell proliferation in human cancer cells

Four PC cell lines PANC-1, MIAPaCa-2, CFPAC-1 and BxPC3 grew more rapidly (Fig 4E) when they had lipid and ApoA-II added to their culture medium compared with controls, ApoA-II alone or with lipid alone (except CFPAC-1 cells). Alternatively ApoA-II downregulated normal HPDE6 cells (Fig 4E). This was most evident at 48 h and implies that the combination of lipid and ApoA-II increased the uptake of lipid and thus energy allowing the PC cells to grow more rapidly. There was a similar effect on human cancer cell lines from lung, breast and prostate at 48 h (S4 Fig). However, the ApoA-II did not promote the growth of the breast cancer cell line MCF7, which retained the characteristics of differentiation and grows slowly [40]. In addition, preincubation with anti SR-B1 antibody completely reverse lipid plus ApoA-II stimulated cell proliferation in PANC-1, CFPAC-1 and BXPC3 cells and partially but significantly in MIAPaCa-2 cells (Fig 4E). Interestingly, inhibition of SR-B1 enhanced ApoA-II plus lipid induced downregulation of cell growth in HPDE6 (Fig 4E). Anti SR-B1 alone at 2.5μg/ml had no significant effect on HPDE6, PANC-1, MIAPaCa-2 and BxPC3 cell lines, while significantly downregulated CFPAC-1 cell growth (Fig 4D). This may be due to the difference density level of SR-B1 in PANC-1, MIAPaCa-2, BxPC3 and CFPAC-1 cell lines (Fig 4A).

## Discussion

ApoA-II changed the physical structure of SMOF lipid by reducing the size of the lipid particles and inducing the formation of a bilayer membrane nanoparticle- like structure. This is consistent with the known structure and function of ApoA-II in HDL [41]. The combination of ApoA-II and lipid significantly promoted the growth of PC cell lines and cell lines from lung, breast and prostate cancers. In addition, ApoA-II enhance lipid uptake in PANC-1, CFPAC-1, primary tumour cells and in PDXT. The uptake of exogenous lipid emulsion with ApoA-II into PC tumours may utilize the SR-B1 receptor (Fig 2A4), because it forms a HDL like structure which has a high-affinity to SR-B1 [26]. ApoA-II helps to target lipid to pancreatic cancer in a manner which could be exploited for theranostic purposes.

Apo A-II binds and shrinks a subset of HDL and prolongs its circulation time [12] and inhibits lipoprotein lipase [13]. Pancreatic cancers consume lipids [42] and protein [43] from the extracellular space for the maintenance of anabolic metabolism [44]. Xue A *et al* found that serum ApoA-II is reduced in PC [1] patient sera which may be due to the increase in consumption by PC. In this study, the fluorescent photomicrographs show the uptake of the SMOFlipid to the cytoplasm of the pancreatic cancer cells when added with ApoA-II. This suggests that ApoA-II helps to target lipid nanoparticles into PC cells. One of the possible explanations is ApoA-II may stablise HDL and increased the binding affinity to its receptors, namely, SR-B1 [15], which we have demonstrated to be highly expressed in PC cells and PDAC tumours. We have also demonstrated preferential uptake of SMOFlipid when combined with ApoA-II was greater in xenograft compared to normal mouse pancreas. Although this study used human ApoA-II there was some uptake in the liver and spleen, presumably being attracted to murine SR-B1. Nevertheless, SMOFlipid plus ApoA-II also retained selectively in liver and spleen, well known sites for liposome accumulation [45–47]. This is achieved may be due to the ability of these SMOFlipid plus ApoA-II to circulate with long half-lives (2–4.5 days) (Fig 4, [48, 49]), enabling them repeated passages through blood vessels feeding growing tumor. On the other hand exogenous ApoA-II may bind and shrink a subset of HDL and prolong its circulation time [12] by decreasing hepatic clearance [12] and increasing the affinity to tumours SR-B1[15].

In this study, ApoA-II plus lipid enhanced proliferation of PANC-1, MIAPaCa-2, CFPAC-1 and BxPC3 but downregulated normal pancreatic cells. Pancreatic cancers exist in a unique metabolic environment and are extremely dense with interstitial pressures that can exceed 10-times those observed in normal organs like the liver or pancreas [50]. Pancreatic cancer often grows within a fibrotic, abundant, and poorly perfused stroma [51] leading to limited access to nutrients and oxygen. The increased expression of SR-B1 may be an adaptation to facilitate survival and growth by the ability to scavenge lipid and recycled metabolic substrates. This adaptation may be able to be utilized for theranostic purposes to improve the management of patients with pancreatic cancer.

## Supporting Information

S1 Fig Phase transition interaction of ApoA-II and lipid emulsion. (TIF)

S2 Fig **Enhanced lipid uptake by ApoA-II in PANC-1 pancreatic cancer cell line** (A), PDAC primary cells (B) and A549 lung cancer cell line (C) by Confocal imaging.
(TIF)

S3 Fig (A) Sequential spectral reflectance fluorescence images of third generation tumor-bearing mice. Note reflectance of food particles in panel (i) pre-injection which is also evident in the stomach in panel (iv) but the tumour was still seen. Mice were imaged immediately after injection (ii), at 4 h (iii), at 24 h (iv) and at 48 h (v) of lipid with DiD and ApoA-II. Representative tumours were harvested and imaged at 24 h and 48 h as indicated in (vi). (B) Photomicrographs (upper panel) and spectral reflectance fluorescence images (lower panel) of organs at 48 h after tail vein injection of lipid without or with ApoA-II.
(TIF)

S4 Fig Effects of ApoA-II on lipid induced lung cancer cells (A549), breast cancer cells (T47D) and prostate cancer cells (PC3) growth. Values are mean ± standard deviation (SD); *n* = 4. *P < 0.05 versus control, ^‡^*P* < 0.05 versus ApoA-II; ^#^*P* < 0.05 versus lipid-treated cells, with the use of analysis of variance.
(TIF)
