# Supplementary figures and images for: Apolipoprotein A-II Plus Lipid Emulsion Enhance Cell Growth via SR-B1 and Target Pancreatic Cancer In Vitro and In Vivo
Source: PLoS One. 2016 Mar 22;11(3):e0151475. doi: 10.1371/journal.pone.0151475 (PMC4803224; doi:10.1371/journal.pone.0151475)

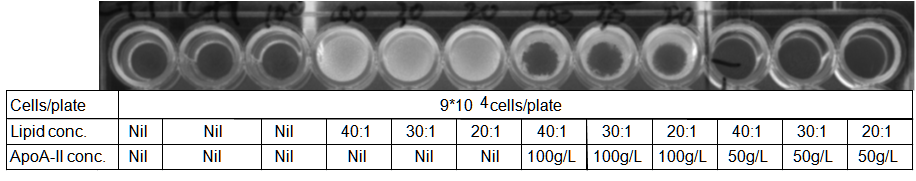

Supplement: S1 Fig — (TIF) [file pone.0151475.s001.tif]

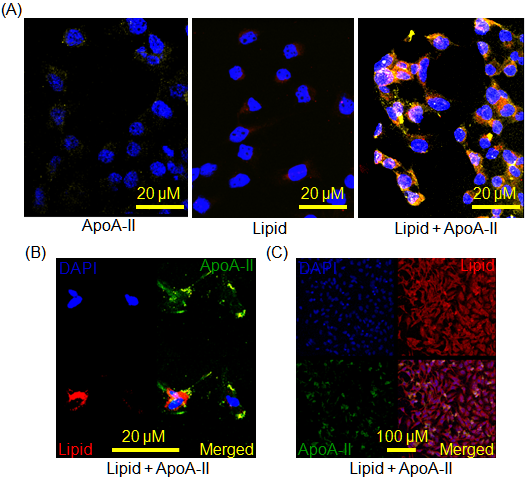

Supplement: S2 Fig — Enhanced lipid uptake by ApoA-II in PANC-1 pancreatic cancer cell line (A), PDAC primary cells (B) and A549 lung cancer cell line (C) by Confocal imaging. (TIF) [file pone.0151475.s002.tif]

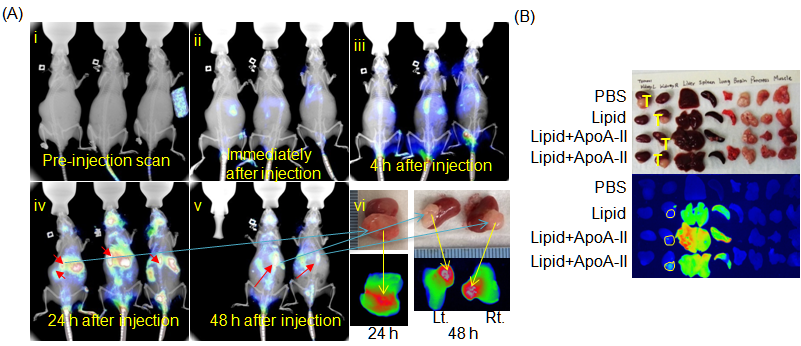

Supplement: S3 Fig — (A) Sequential spectral reflectance fluorescence images of third generation tumor-bearing mice. Note reflectance of food particles in panel (i) pre-injection which is also evident in the stomach in panel (iv) but the tumour was still seen. Mice were imaged immediately after injection (ii), at 4 h (iii), at 24 h (iv) and at 48 h (v) of lipid with DiD and ApoA-II. Representative tumours were harvested and imaged at 24 h and 48 h as indicated in (vi). (B) Photomicrographs (upper panel) and spectral reflectance fluorescence images (lower panel) of organs at 48 h after tail vein injection of lipid without or with ApoA-II. (TIF) [file pone.0151475.s003.tif]

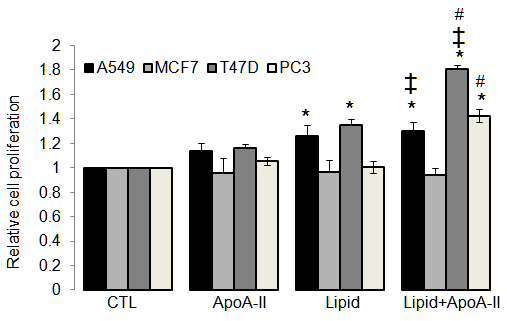

Supplement: S4 Fig — Values are mean ± standard deviation (SD); n = 4. *P < 0.05 versus control, ‡P < 0.05 versus ApoA-II; #P < 0.05 versus lipid-treated cells, with the use of analysis of variance. (TIF) [file pone.0151475.s004.tif]
